# Supplementary material for: Quantification of Pork, Chicken, Beef, and Sheep Contents in Meat Products Using Duplex Real-Time PCR
Source: Foods. 2023 Aug 7;12(15):2971. doi: 10.3390/foods12152971 (PMC10418471; doi:10.3390/foods12152971)
Supplement: Supplementary file 1 [file foods-12-02971-s001.zip › foods-2465587-supplementary.pdf]

Quantification of Pork, Chicken, Beef and Sheep Contents in Meat Products Using Duplex Real-Time PCR

Supplementary Materials

Table S1. Composition of PT samples.

Sample name	Sample Description	Date of Distribution
FAPAS-PT1	The test material is raw lamb (94%) (freeze-dried) and has been contaminated with goat (3%) and pork (3%).	7.2022
FAPAS-PT2	The test material is raw beef (90%) (freeze-dried) and has been contaminated with equine (5%) (horse) and lamb (5%).	2.2022
FAPAS-PT3	The test material is raw chicken (92%) (freeze-dried) and has been contaminated with pork (3%) and turkey (5%).	4.2021

FAPAS: The Food Analysis Performance Assessment Scheme

Table S2. Specificity of the real-time PCR system.

Species	Expected results					Tested results				
	Pork	Chicken	Beef	Sheep	PC	Pork	Chicken	Beef	Sheep	PC
Cattle (*Bos taurus*)	ND	ND	D	ND	D	ND	ND	D	ND	D
Chicken (*Gallus gallus*)	ND	D	ND	ND	D	ND	D	ND	ND	D
Crocodile (*Crocodylus niloticus*)	ND	ND	ND	ND	D	ND	ND	ND	ND	D
Donkey (*Equus asinus*)	ND	ND	ND	ND	D	ND	ND	ND	ND	D
Duck (*Anatidae*)	ND	ND	ND	ND	D	ND	ND	ND	ND	D
Goat (*Capra hircus*)	ND	ND	ND	ND	D	ND	ND	ND	ND	D
Goose (*Anserinae*)	ND	ND	ND	ND	D	ND	ND	ND	ND	D
Hare (*Lepus europaeus*)	ND	ND	ND	ND	D	ND	ND	ND	ND	D
Horse (*Equus caballus*)	ND	ND	ND	ND	D	ND	ND	ND	ND	D
Kangaroo (*Macropodidae*)	ND	ND	ND	ND	D	ND	ND	ND	ND	D
Ostrich (*Struthio camelus*)	ND	ND	ND	ND	D	ND	ND	ND	ND	D
Pig (*Sus scrofa domestica*)	D	ND	ND	ND	D	D	ND	ND	ND	D
Rabbit (*Oryctolagus cuniculus*)	ND	ND	ND	ND	D	ND	ND	ND	ND	D
Red deer (*Cervus elaphus*)	ND	ND	ND	ND	D	ND	ND	ND	ND	D
Roe deer (*Capreolus capreolus*)	ND	ND	ND	ND	D	ND	ND	ND	ND	D
Sheep (*Ovis aries*)	ND	ND	ND	D	D	ND	ND	ND	D	D
Turkey (*Meleagris gallopavo*)	ND	ND	ND	ND	D	ND	ND	ND	ND	D
Wild boar (*Sus scrofa scrofa*)	D	ND	ND	ND	D	D	ND	ND	ND	D
Black mustard (*Brassica nigra*)	ND	ND	ND	ND	D	ND	ND	ND	ND	D
Broccoli (*Brassica oleracea*)	ND	ND	ND	ND	D	ND	ND	ND	ND	D
Carrot (*Daucus carota*)	ND	ND	ND	ND	D	ND	ND	ND	ND	D
Celery (*Apium graveolens*)	ND	ND	ND	ND	D	ND	ND	ND	ND	D
chili pepper (*Capsicum sp.*)	ND	ND	ND	ND	D	ND	ND	ND	ND	D
garlic (*Allium sativum*)	ND	ND	ND	ND	D	ND	ND	ND	ND	D
ginger (*Zingiber officinale*)	ND	ND	ND	ND	D	ND	ND	ND	ND	D
Kailan (*Brassica oleracea var. alboglabra*)	ND	ND	ND	ND	D	ND	ND	ND	ND	D
onion (*Allium cepa*)	ND	ND	ND	ND	D	ND	ND	ND	ND	D
parsley (*Petroselinum crispum*)	ND	ND	ND	ND	D	ND	ND	ND	ND	D
rapeseed (*Brassica napus*)	ND	ND	ND	ND	D	ND	ND	ND	ND	D
rice (*Oryza sativa*)	ND	ND	ND	ND	D	ND	ND	ND	ND	D
rosemary (*Rosmarinus officinalis*)	ND	ND	ND	ND	D	ND	ND	ND	ND	D
rye (*Secale cereale*)	ND	ND	ND	ND	D	ND	ND	ND	ND	D
tomato (*Solanum lycopersicum*)	ND	ND	ND	ND	D	ND	ND	ND	ND	D
wheat (*Triticum aestivum*)	ND	ND	ND	ND	D	ND	ND	ND	ND	D
cabbage (*B rassica oleracea var. oleracea*)	ND	ND	ND	ND	D	ND	ND	ND	ND	D
shiitake mushroom (*Lentinula edodes*)	ND	ND	ND	ND	D	ND	ND	ND	ND	D

PC: internal positive amplification control on the 18S rRNA gene; ND: not detected. The genomic DNA for cattle (*Bos taurus*), chicken (*Gallus gallus*), pig (*Sus scrofa domestica*), sheep (*Ovis aries*) and duck (*Anatidae*) were extracted using the DNeasy Blood & Tissue Kit (Qiagen, Hilden, Germany); genomic DNA from other common food ingredients: black mustard (*Brassica nigra*), broccoli (*Brassica oleracea*), carrot (*Daucus carota*), celery (*Apium graveolens*), chili pepper (*Capsicum sp.*), garlic (*Allium sativum*), ginger (*Zingiber officinale*), Kailan (*Brassica oleracea var. alboglabra*), onion (*Allium cepa*), parsley (*Petroselinum crispum*), rapeseed (*Brassica napus*), rice (*Oryza sativa*), rosemary (*Rosmarinus officinalis*), rye (*Secale cereale*), tomato (*Solanum lycopersicum*), wheat (*Triticum aestivum*), cabbage (*B rassica oleracea var. oleracea*), and shiitake mushroom (*Lentinula edodes*) were extracted using CTAB method described in section 2.2; the genomic DNA solution for crocodile (*Crocodylus niloticus*), donkey (*Equus asinus*), goat (*Capra hircus*), goose (*Anserinae*), hare (*Lepus europaeus*), horse (*Equus caballus*), kangaroo (*Macropodidae*), ostrich (*Struthio camelus*), rabbit (*Oryctolagus cuniculus*), red deer (*Cervus elaphus*), roe deer (*Capreolus capreolus*), turkey (*Meleagris gallopavo*) and wild boar (*Sus scrofa scrofa*) were purchased from Eurofins Scientific (France).

Table S3. Performance comparison of our duplex qPCR assays to other reported method.

Parameters	Duplex qPCR developed in this study	Multiplex qPCR reported by Köppel R.et al. 2013 [24]	Multiplex qPCR reported by Köppel R.et al. 2020 [25]
Amplification efficiencies %	98.7 - 104.5	59 - 107	89 - 97
Correlation R^2	0.99 - 1.0	0.96 - 0.98	0.98 - 1.0
Precision ± %	2.45 – 24.58	15 - 24	4.09-103.23
Accuracy ± %	0.14 – 24.07	4.1 - 167.7	1.0 – 102.1

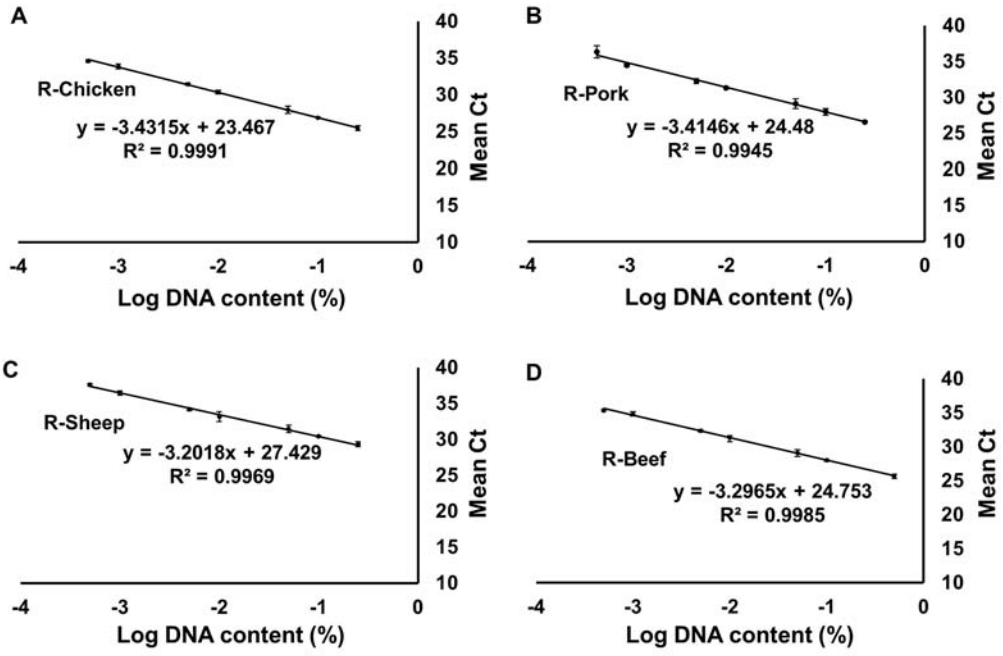

Figure S1. Standard curves plotted by the mean Ct values against 7 dilution series (25%, 10%, 5%, 1%, 0.5%, 0.1% and 0.05%) using singplex qPCR for chicken (A), pork (B), sheep (C) and beef (D). A DNA mixture consisting of 25% pork, 25% chicken, 25% beef and 25% sheep (20 ng/μL) was serially diluted in herring sperm DNA solution (20 ng/μL) and analyzed in 6 replicates.

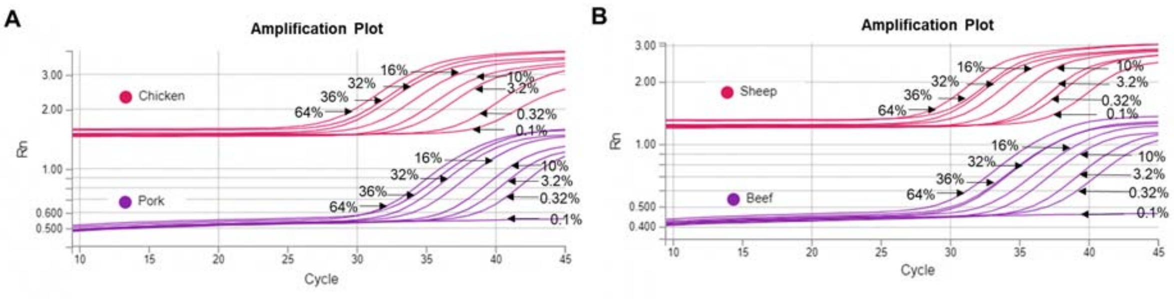

Figure S2. The real-time PCR data of amplification curves for boiled meat using serial diluted DNA. (A) Amplification curves for boiled chicken and pork; (B) Amplification curves for boiled beef and sheep. For serially diluted DNA, 0.32 ng of DNA were detected for all boiled meat species.
